# First-Bite Syndrome: A Rare Complication of Carotid Endarterectomy

**DOI:** 10.7759/cureus.15868

**Published:** 2021-06-23

**Authors:** Andras Bikk, Sohrab Sohrabi, Prashanth Navaran, Cameron Farsar

**Affiliations:** 1 Surgery, VA Central California Health Care System, Fresno, USA; 2 Medical Education, Idaho College of Osteopathic Medicine, Meridian, USA

**Keywords:** carotid endarterectomy, first bite syndrome, postoperative pain, chewing, eating

## Abstract

This report describes the rare complication of first-bite syndrome (FBS) after carotid endarterectomy (CEA). Although FBS is well known in otolaryngologic literature, it is rarely discussed in relation to vascular surgery. FBS is most commonly a postoperative pain syndrome that is thought to be the result of selective parotid gland sympathetic denervation. The resultant facial pain is centered around the parotid region and triggered by initiation of mastication. The pain is severe, but short in duration, and quite specific in pattern.

We present a case of FBS after CEA with complex anatomy. The patient developed typical symptoms of ipsilateral parotid, mandibular pain during the postoperative course. Workup excluded other diagnoses. The symptoms were self-limiting but did not resolve completely. Vascular surgeons, who are universally aware of the presentation of Horner’s syndrome, should also be aware of this rare complication with similar pathophysiology.

## Introduction

The original use of the term “first-bite syndrome” (FBS) is connected to a gastroenterologist Dr. Haubrich [1]. In 1986, he described recurrent episodes of dysphagia, which was associated with retrosternal pain in his patients. He attributed the symptoms to esophageal spasm. While this information is noted here for historical completeness, what we describe as FBS today is completely unrelated to this. The first description in the literature of the herein discussed syndrome appeared in Gardner and Abdullah’s neurosurgery report as a complication of superior cervical ganglion excision in 1955 [2]. This operation was used as a therapeutic procedure for variable diseases at the time. In 1988, Netterville, an otolaryngologist, noted the syndrome described by Gardner on his postoperative patients who underwent skull base vagal paraganglioma resections. In his report, he named the complication FBS, which remains in effect today [3]. Remarkably, both Gardner and Netterville speculated that the symptoms were related to a problem with the parotid gland‘s postganglionic sympathetic innervation.

Although there are potentially many causes of pain in the head and neck region (e.g., Eagle’s syndrome, glossopharyngeal neuralgia, trigeminal neuralgia, temporomandibular joint arthralgia, infection, and malignancy), none of them share the distinguishing characteristic presentation of FBS. The symptoms typically present between the fifth and seventh postoperative day in the form of severe, sharp pain in the parotid gland, pharynx, and mandible, at the first bite of food. The pain only lasts for seconds, then rapidly resolves despite continued mastication. This pain pattern recurs with each meal.

The anatomic and pathophysiologic theories behind FBS are relatively complex and related specifically to the parapharyngeal space (PPS). The PPS is a tight upside-down pyramidal space bordered by the skull base superiorly, the hyoid bone inferiorly, the mandibular ramus and parotid gland laterally, and finally the pharynx medially. It contains the internal carotid artery (ICA), the internal jugular vein, cranial nerves (IX, X, XI, XII), lymph nodes, and the cervical sympathetic chain with the superior cervical ganglion (SCG) and its postganglionic fibers. The SCG lies posterior to the carotid artery. It is approximately 3 cm long and the postganglionic fibers run along the external carotid artery (ECA) and the ICA. It is important to note that the fibers next to the ECA innervate the parotid gland and the sweat glands on the face. Fibers along the ICA innervate the eyelid, the pupil, and the sweat glands of the forehead. Postganglionic sympathetic fiber disruption adjacent to the ICA or SCG and main sympathetic trunk injury causes Horner’s-type symptoms. However, in the latter case, the remaining residual autonomous activity of the SCG may prevent the development of FBS. Selective disruption of those postganglionic fibers that innervate the parotid gland are necessary for FBS to appear [4,5].

FBS can develop after carotid surgery, but it is rarely reported. We present our case of FBS that developed after elective carotid endarterectomy (CEA) to alert vascular surgeons to the unique presentation, pathophysiology, and available treatment options of this rare surgical complication. To our knowledge, this report is only the eighth presentation of FBS in the literature related to CEA [6-12].

## Case presentation

**Figure 1:**
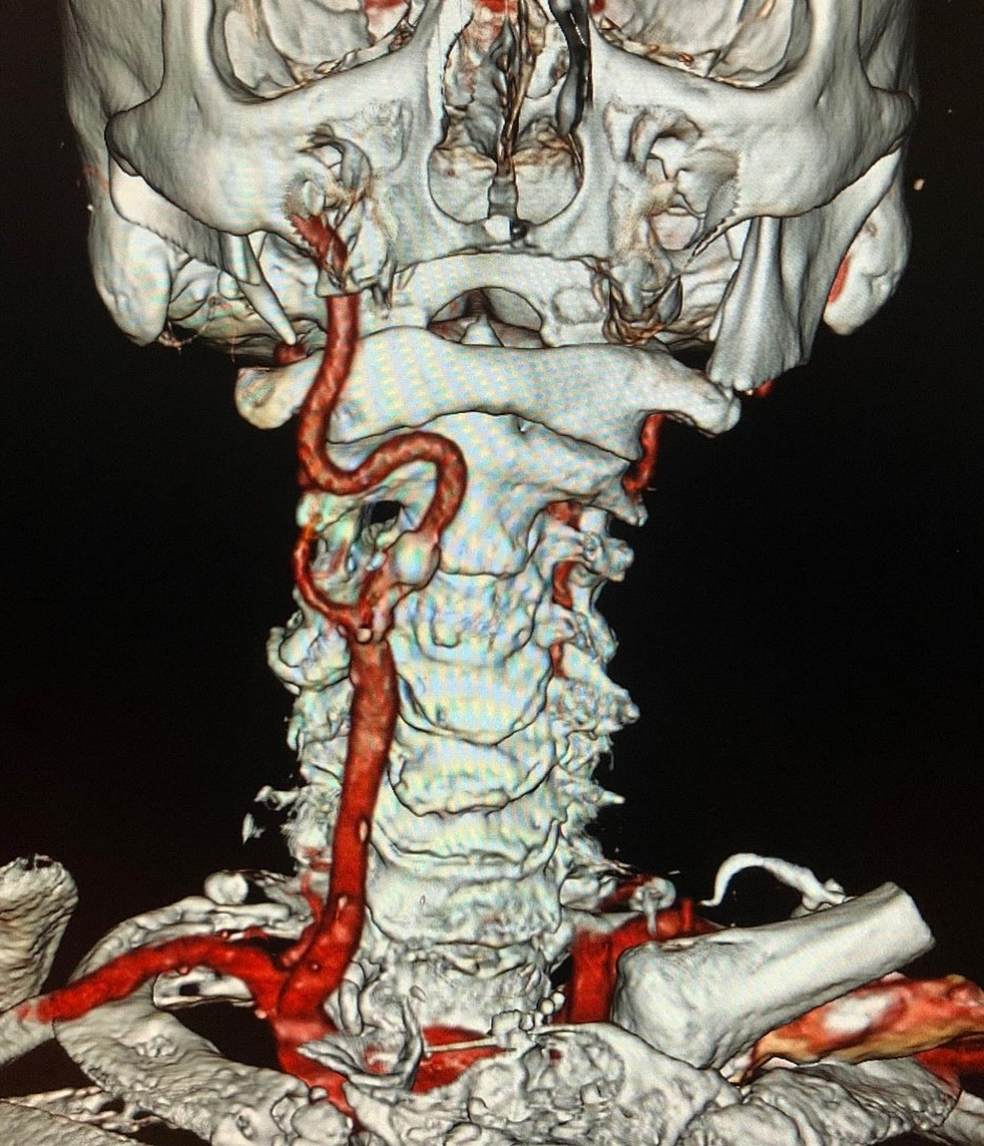
Right carotid artery with tortuosity

An 80-year-old male who previously underwent a left CEA developed pulsatile tinnitus in his right ear. Computed tomography angiogram showed occlusion of the left ICA. In the right ICA, a focal, circumferentially calcified plaque was found, which was causing 80% stenosis (Figure 1).

The carotid bifurcation was relatively high, and the ICA had severe tortuosity, displacing the artery behind the pharynx. The aortic arch was significantly calcified as well. Based on the aortic arch and the plaque quality, the patient was a suboptimal candidate for trans-femoral carotid stent placement, hence a right CEA was performed. The ICA was shortened and straightened with posterior plication and closed with bovine pericardial patch.

On the seventh postoperative day, the patient began to complain of sudden-onset, severe, sharp pain at the right mandibular angle and parotid region. The pain consistently occurred at the first bite of food, lasted for seconds, and rapidly resolved as the chewing continued. The pain returned later with each meal in the same pattern. Physical examination and reimaging did not find any abnormality. Based on the recurrent typical symptoms, and through diagnosis of exclusion, FBS was diagnosed. The short-lived pain severity improved gradually to a tolerable level, and no specific treatment or intervention was initiated. During our more than two-year follow-up, the right ICA remained patent without restenosis. However, the patient’s symptoms have not completely resolved. As the patient phrased it he “learned to live with it” and declined interventions for symptom relief.

## Discussion

FBS is a rare, chronic, postoperative pain syndrome of the head and neck. It is most commonly a complication of surgery in the PPS, infratemporal fossa, or on the deep lobe of the parotid gland itself. Although CEA requires limited intrusion into these spaces, the dissection of a tortuous or misplaced ICA may force the surgeon to dissect deeper into the PPS or place retractors higher in the space applying pressure to the deep parotid lobe laterally. This may result in traction injury or disruption of the delicate postganglionic sympathetic fibers along the ECA or close to the deep parotid lobe that they innervate. The parasympathetic innervation to the parotid is not interrupted with dissection in the PPS. It follows a completely different pathway through the otic ganglion at the skull base to the auriculotemporal nerve.

Once the postganglionic sympathetic fibers are damaged, the unopposed parasympathetic stimulus to the parotid’s myoepithelial cells is thought to be the cause of the intense pain with the start of each meal. This is similar to the pathophysiology of the Horner’s-type symptoms, which are also caused by the imbalance between the sympathetic/parasympathetic system for the dominance of the latter. FBS presenting without Horner’s symptoms appears to represent the result of a selective parotid sympathectomy that caused denervation hypersensitivity.

The natural course of FBS is relatively benign. Spontaneous resolution of the symptoms is relatively common within 6-12 months, making a conservative treatment approach compelling. In a series, complete resolution was reported in 12%, some improvement in 69%, and no improvement only in 15% of the cases [13]. In a review, Laccourreye et al. summarized several treatment options [14]. Injection of botulinum toxin into the painful zone of the parotid gland appears to be the most effective option, and may last several months [15,16]. Although radiation therapy was effective in oncologic patients [17], it appears to be excessive under these benign conditions.

Avoiding excessive ECA and PPS dissection or retraction that causes traction in the PPS is prudent in carotid surgery and likely the best way to avoid the complication. In recent years, trans-carotid artery revascularization was introduced to the surgical armamentarium with good early results [18]. As this technique does not require dissection in the PPS, it can lower the risk of the development of FBS in properly selected patients. The technique could be especially useful in patients with significant deviation of the carotid artery toward, or behind, the pharynx where during a CEA more extensive dissection and PPS retraction may become necessary.

## Conclusions

FBS is a rare complication of CEA, which is most likely the result of selective parotid sympathectomy. Excessive retraction and dissection in the PPS or along the ECA is the potential cause of postganglionic fiber disruption and the resultant denervation hypersensitivity. The diagnosis can be secured by the typical presentation and exclusion of other regional and postoperative problems. It has a relatively benign course and the symptoms often improve spontaneously. Should debilitating symptoms occur, botulinum toxin injection into the parotid gland appears to be reasonable and effective.
